# 3D Bioprinting Strategies, Challenges, and Opportunities to Model the Lung Tissue Microenvironment and Its Function

**DOI:** 10.3389/fbioe.2021.773511

**Published:** 2021-11-24

**Authors:** Mabel Barreiro Carpio, Mohammadhossein Dabaghi, Julia Ungureanu, Martin R. Kolb, Jeremy A. Hirota, Jose Manuel Moran-Mirabal

**Affiliations:** ^1^ Department of Chemistry and Chemical Biology, McMaster University, Hamilton, ON, Canada; ^2^ Firestone Institute for Respiratory Health, Division of Respirology, Department of Medicine, McMaster University, Hamilton, ON, Canada; ^3^ School of Biomedical Engineering, McMaster University, Hamilton, ON, Canada; ^4^ McMaster Immunology Research Centre, Department of Pathology and Molecular Medicine, McMaster University, Hamilton, ON, Canada; ^5^ Division of Respiratory Medicine, Department of Medicine, University of British Columbia, Vancouver, BC, Canada; ^6^ Department of Biology, University of Waterloo, Waterloo, ON, Canada; ^7^ Centre for Advanced Light Microscopy, McMaster University, Hamilton, ON, Canada

**Keywords:** assisted bioprinting, bioink, biomaterial ink, alveolus, biomimetic, extracellular matrix, additive manufacturing

## Abstract

Human lungs are organs with an intricate hierarchical structure and complex composition; lungs also present heterogeneous mechanical properties that impose dynamic stress on different tissue components during the process of breathing. These physiological characteristics combined create a system that is challenging to model *in vitro*. Many efforts have been dedicated to develop reliable models that afford a better understanding of the structure of the lung and to study cell dynamics, disease evolution, and drug pharmacodynamics and pharmacokinetics in the lung. This review presents methodologies used to develop lung tissue models, highlighting their advantages and current limitations, focusing on 3D bioprinting as a promising set of technologies that can address current challenges. 3D bioprinting can be used to create 3D structures that are key to bridging the gap between current cell culture methods and living tissues. Thus, 3D bioprinting can produce lung tissue biomimetics that can be used to develop *in vitro* models and could eventually produce functional tissue for transplantation. Yet, printing functional synthetic tissues that recreate lung structure and function is still beyond the current capabilities of 3D bioprinting technology. Here, the current state of 3D bioprinting is described with a focus on key strategies that can be used to exploit the potential that this technology has to offer. Despite today’s limitations, results show that 3D bioprinting has unexplored potential that may be accessible by optimizing bioink composition and looking at the printing process through a holistic and creative lens.

## Introduction

### The Lung and Respiratory Health

Lungs are large organs located in the thoracic cavity, and the primary organs of the human respiratory system. Every human breathes over 10,000 L of air each day to obtain the oxygen needed to survive (Huff, Carlsten, and Hirota 2019). During this necessary process, the lungs are exposed to potential insults including pathogens, allergens, air pollution, and tobacco smoke. These environment and individual experiences can have an impact on the genetic material and contribute to the development of chronic lung disease. The World Health Organization Global Burden of disease Report highlights that pulmonary diseases and respiratory tract infections are among the top five causes of mortality in humans (Roth et al., 2018). The end result of many chronic lung diseases is irreversible loss of lung function, reduced gas exchange, and poor quality of life. Humans are not able to regenerate lung tissue and transplants are the most common restorative intervention for individuals with end-stage chronic lung diseases (Swaminathan et al., 2020). Lung transplant procedures are not keeping up with demand despite increased availability due to improved donor-recipient matching and maintenance of donor lung viability (Thabut and Mal 2017). Successful transplant matches are still susceptible to rejection and rejection-related complications that cause high morbidity and mortality following transplantation (Thabut and Mal 2017). Tissue engineering can provide tools to build reliable models that lead to a better understanding of lung structure and function at the cellular and tissue level (An et al., 2015; Yan et al., 2018; Zhao et al., 2018), and has the long-term potential to address the demand for transplantable synthetic lung tissue.

**FIGURE 1:**
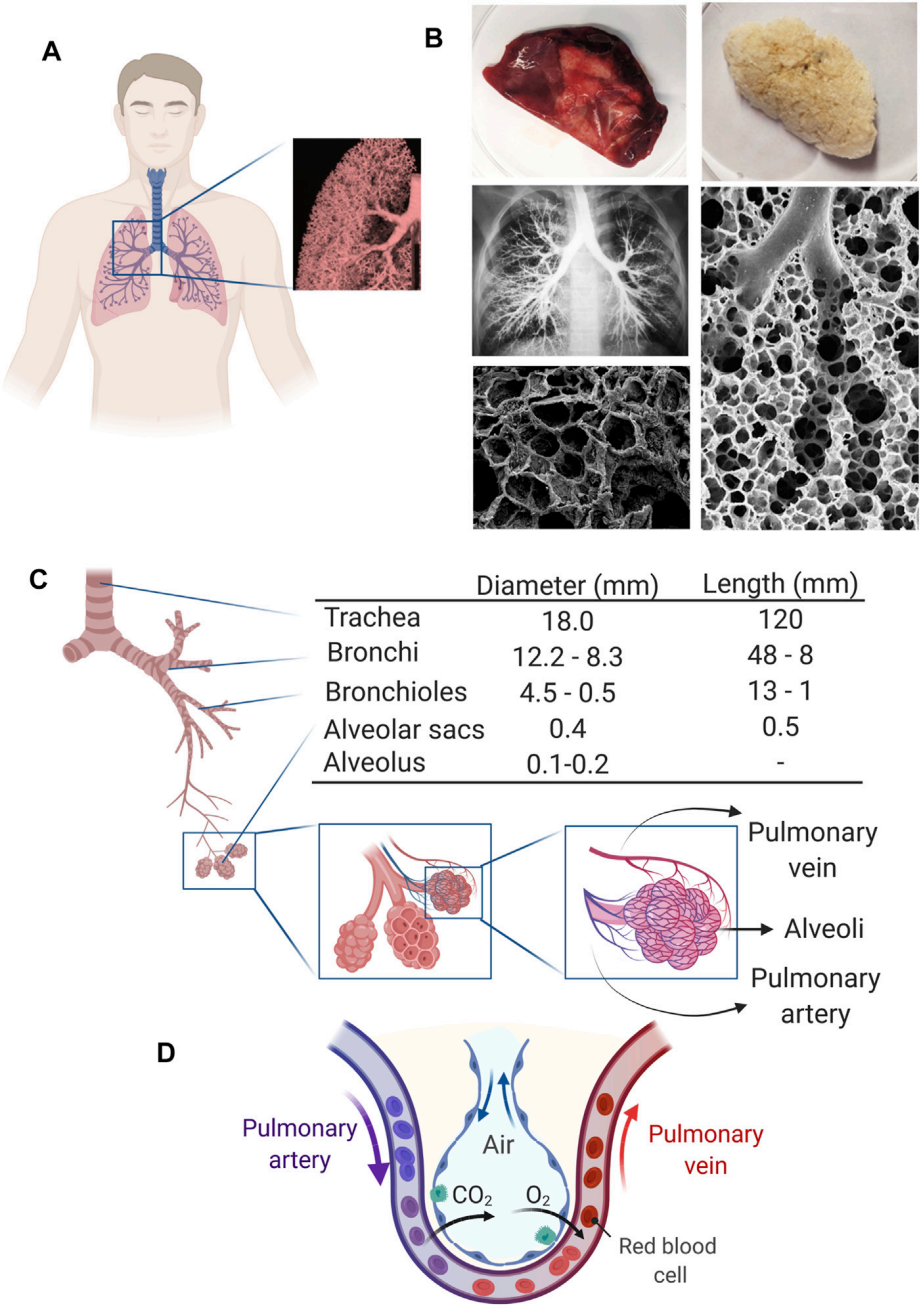
Human lung structure and function. **(A)** Representation of the lung in the human body and its appearance. Inset: Pulmonary tissue contains many interconnected airways that give the lung its spongy structure. **(B)** Macroscopic images of native and decellularized human lungs (Reproduced from reference (Booth et al., 2012)) along with images that show the internal structure of lung tissue (Reproduced from references (Freed et al., 2012; Weibel 2013)). **(C)** Representation of the respiratory tree and the airway branching of the human lung and its approximate dimensions. Dimensions for all structures are reported for the resting lung, while for the alveolus, a range for resting and expanded lung is reported (Data retrieved from references (Bouhuys 1977; Bajaj et al., 2016) and (Ochs and Nyengaard. 2004)). **(D)** Schematic representation of the gas exchange occurring at the alveolus vascular interface.

In nature and particularly in multicellular organisms, function arises from structure, and the lung is no exception. The human lung presents a complex architecture consisting of the airways that function as conduits and lung parenchyma and alveoli that are responsible for gas exchange. The internal structure of the lungs is highly vascularized and presents intricate passages formed by bronchi and bronchiole that bifurcate from the trachea and end in the alveoli sacs (Figure 1). Alveoli regions occupy ∼65% of the lung surface, and their membrane is key in the gas exchange process (Stone et al., 1992). The lung presents over 60 different kinds of cells that perform a wide variety of functions (Stone et al., 1992; Franks et al., 2008; Bajaj et al., 2016). These cells are supported by and distributed within the extracellular matrix (ECM), which constitutes almost 50% of the nonalveolar tissue in the lung.

To design an accurate *in vitro* lung model, we should identify the main tissue components and understand their function in healthy and diseased organs. Relevant cells should also be selected based on the final application of the pulmonary model. Among the many different cells that reside in the lung, epithelial cells are the predominant cell type. Epithelial cells cover the entire surface of airways and alveoli, and exist as different subtypes based on their roles. Ciliated, club, and goblet cells are the primary epithelial cells at the conduction zone (the upper respiratory tract), where the thickness of a healthy epithelium ranges from 25 to 40 µm (Xie et al., 2018). The epithelium thickness reduces down the airway, with club cells dominating over goblet cells in the small bronchioles. Club cells have a cuboidal morphology and contain fewer ciliated subpopulations (Kuzovlev, Shabanov, and Grechko 2019). Upon reaching the respiratory zone of the lung, the terminal bronchioles end in alveolar sacs, which consist of alveolar type 1 (AT1) cells and alveolar type 2 (AT2) cells. Vascularized endothelium is adjacent to the alveolar sacs and is responsible for bringing blood close to the alveoli for gas exchange. AT1 cells are ultra-thin and flat and play a central role in gas exchange. Although AT1 cells are not the main cellular population in the lung parenchyma (less than ∼11%), they cover more than 90% of the lung’s surface area (Bonner 2008). In contrast, AT2 cells form a larger cell population of the lung parenchyma (12–16%); however, they cover only around 7% of the lung’s surface area (Bonner 2008). This is because AT2 cells do not contribute to gas transfer and their primary function is to produce surfactant, which is a complex phospholipid- and protein-based substance for reducing surface tension at the air-liquid interface in alveolar sacs (Dobbie 1996). In addition, AT2 cells can differentiate into AT1 cells (serving as alveolar stem cells) when the lining of AT1 cells is damaged and needs to be repaired (Nguyen et al., 2019). Fibroblasts are another important lung cell type that is largely responsible for the deposition of ECM, which provides structure on a macroscopic level and surfaces for cell adhesion and proflieration at the micro-to-nanoscopic level (Upagupta et al., 2018).

The physical and biochemical properties of bioprinted constructs should resemble the natural microenvironment in the lung to enable cell differentiation and proper function. To achieve such a goal, it is necessary to know the key components of the lung’s ECM and their contributions to cellular function. The lung ECM is dominated in composition by collagen, elastin, fibronectin, glycoproteins, and proteoglycans, that collectively constitute the structural scaffold with the elastic biomechanical properties required for the repeated ventilation cycles over a human lifespan (Zhou et al., 2018). Collagen types I and III are the primary components of the lung ECM providing the required tensile strength. However, the alveolar sacs and basement membranes, where gas exchange occurs, are mainly composed of collagen type IV and laminin. Elastin is the ECM protein that overall provides needed elasticity to the lung. The ECM is not a fixed and static support, but rather an evolving dynamic structure where the composition and properties vary depending on cell types present in the local microenvironment and their metabolic processes (Zhou et al., 2018). Furthermore, the ECM modulates cell behavior and plays a key role in disease evolution and injury repair processes (Zhou et al., 2018; Upagupta, Yanagihara, and Kolb 2020).

It has been reported that the elastic modulus (stiffness) of a healthy human lung is ∼2.0 ± 0.1 kPa, and that the lung tissue stiffness can be changed by various diseases (Booth et al., 2012). For instance, the stiffness of the lung tissue increases up to 17 ± 2 kPa in idiopathic pulmonary fibrosis (IPF), an interstitial lung disease that is characterized by the excessive deposition of ECM proteins in the lung (Yanagihara et al., 2020). Fibroblasts are a major cell responsible for pathology in IPF, and are able to transition to myofibroblasts that continuously produce and secrete fibrillar collagen-rich ECM, thereby increasing the stiffness of the lung tissue (Pakshir and Hinz 2018). In lung cancer, the interaction between cancerous cells and the ECM is likely to share similarities with IPF. These examples highlight the importance of bioink formulations in the bioprinting process, which should mimic the mechanical and biochemical properties of healthy or diseased lung (depending on the objective of the study) because they influence cellular functionality and fate (Hu et al., 2021).

The constant interaction of the lung with the external environment requires an immune system capable of recognizing threats and responding accordingly (Huff, Carlsten, and Hirota 2019). The epithelium is the first cellular line of defense that protects the conducting airways during ventilation. Airway epithelial cells secrete mucus and airway surface lining fluid that can trap and contain potential insults, followed by directional removal in the proximal direction in the lungs via the mucociliary ladder (Bajaj et al., 2016; Huff, Carlsten, and Hirota 2019). Epithelial cells interact with a basement membrane of ECM that is also home to smooth muscle cells, fibroblasts, and resident immune cell populations. The accessibility of the lungs has been leveraged for therapies of chronic lung disease through inhalation delivery routes of bronchodilators and anti-inflammatory drugs (Roth et al., 2018; Huff, Carlsten, and Hirota 2019). The non-invasive inhalation method has also been explored for the delivery of drugs targeting other organs (Patton and Byron 2007; Olsson et al., 2011). The urgent need for effective solutions for patients with pulmonary diseases and the advantages of the pulmonary route for delivering drugs with low oral availability are strong motivators to develop tools that allow a better understanding of the lung and lead to the development of effective treatments in shorter time frames.

Recreating the human lung complexity is a challenging task with different levels of complexity (Nichols et al., 2014; Bajaj et al., 2016). Reproducing the main geometrical features of the lung with appropriate dimensions is the first step to recreate the natural environment of the cells and create the basis to address the lung function (Itoh, Nishino, and Hatabu 2004; Bajaj et al., 2016). Micro and macro composition of structure is another crucial factor because it will determine the biochemical and mechanical properties of the scaffold, and both aspects will have a combined effect on how cells behave (Nichols et al., 2014; Niemeyer et al., 2018; Zhou et al., 2018; Doryab et al., 2019). Last but not least, it is important to use biologically relevant cell types deposited in a careful and specific manner inside the structure. Each of the mentioned factors has its challenges that need to be overcome to create an appropriate model.

This review focuses on 3D bioprinting, a technology that offers a unique combination of capabilities to mimic lung tissue structure. To account for the state of the art, a summary of the current models developed to study the lung is presented in this review, discussing advantages and limitations. We also describe the strengths of 3D bioprinting and how this technology can help to address current model limitations. Yet, 3D bioprinting has its own inherent limitations, which are highlighted along with a detailed description of strategies that allow to overcome them. Finally, a summary of how 3D bioprinting techniques have been used so far to create lung tissue models is described along with the gaps that need to be addressed to unlock the full potential of this emerging technology.

### Models to Study the Lung

The study of the physiology and pathology of diseases of the human lung, as well as the study of the pharmacodynamics, pharmacokinetics and toxicology of drugs rely almost completely on the use of model systems. This is due to the inherent risks and ethical considerations that limit direct studies in humans or human organs. In this regard, an ideal model should reproduce the phenomenon being studied in a simple, accurate and robust way, and allow access to information that can lead to trustworthy conclusions. Satisfying all these requirements for lung models is not an easy task, especially when trying to recreate diseases that change the composition and the mechanical properties of the whole organ, where changes occur at the macroscopic and microscopic level and generate local differences, as is the case for emphysema of fibrosis (Popper 2016; Swaminathan et al., 2020; Tsuchiya et al., 2020). While many approaches have been developed to mimic the complex composition and microarchitecture of the lung, this task remains challenging with current technologies (Tsuchiya et al., 2020).

Animal models are extensively used as an accurate and reliable tool to determine toxicity in regulatory protocols (Hartung and Rovida 2009). However, differences in lung anatomy, cell biology and immunity in animals contribute to incomplete modeling of human lung diseases or the pharmacokinetics and pharmacodynamics of drugs (Hal and Ghoshal 1988; Lee et al., 2005; Canning and Chou 2008; Ware 2008; WorpBart van der et al., 2010). In addition to the technical limitations of animal models, the need to reduce costs and time in the drug development process (Lee et al., 2005), combined with the increment of regulatory pressure to ban animal use or at least implement the 3R´s philosophy (Reduce Refine Replace, 2010) have resulted in an urgency to develop reliable *in vitro* models that reproduce the human lung microenvironment.

Two dimensional (2D*) in vitro* monocultures of human lung cell populations have provided foundational insights into how drugs, pollutants, growth factors, and immune mediators impact cell biology. 2D systems are simple and easy to use, offer reproducible results, and diverse biological readouts can be obtained (*e.g.,* gene expression, cell proliferation, cell metabolism, mediator release, cell viability) (Minicis et al., 2007). However, 2D cell culture plates are much stiffer than lung tissue, which alters the cellular biological response, 2D *in vitro* models do not include relevant components of the *in vivo* microenvironment of lung tissue, and often omit interactions between cells. To overcome the limitations of 2D systems, *in vitro* lung cell models are evolving to more closely mimic human lung tissue. For example, lung epithelial cell interfaces have evolved from 2D culture of isolated cells (Forbes 2000; Fuchs et al., 2002; Steimer, Haltner, and Lehr 2005) to complex and elegant 3D designs where coculture of multiple cell types is facilitated by a combination of growth media and intricate structures that offer mechanical support (Hermanns et al., 2004; Rothen-Rutishauser et al., 2008; Klein et al., 2013; Nichols et al., 2014). Advances in stem cell biology, tissue engineering, microfluidics and microengineering are also allowing new approaches to design human-derived microtissues and 3D models that better mimic the conditions that individual cells encounter *in situ* in the human lung. Microtissues are scaffold-free 3D structures containing 500–10,000 cells and their application has gone from studies to understand biological systems to full regenerative medicine (Günter et al., 2016). 3D systems allow recreating cell-cell and cell-matrix interactions, as well as cellular migration, adhesion, support and maturation in 3D, as they occur in real tissues. Microtissues, lung organoids and lung-on-a-chip models are among the most used and reliable approaches to study the underlying mechanisms of lung development and disease pathogenesis. Recent advances in these technologies have been extensively described and the interested reader is directed to comprehensive reviews on these topics (Gkatzis et al., 2018; Niemeyer et al., 2018; Mittal et al., 2019; Tian et al., 2020).

The simplest 3D tissue system that can be formed is spheroids, which result from ECM-assisted cellular self-assembly under a physical constraint, such as suspension in a drop-like system. In addition to spheroids, hydrogels and ECM scaffolds have been used to create model 3D cell constructs (Lee et al., 2009). Organoids are the next level of self-assembled constructs, with more complex structures that are formed following mechanisms similar to those that lead to *in vivo* tissue development (Clevers 2016; Yin et al., 2016). Organoids can reproduce several fundamental biological traits, like cellular organization, cell polarization, cell-cell interactions, and some tissue specific functions. However, they lack aspects of natural tissue, like the mechanical cues and irrigation provided by vasculature and a circulating immune system. Thus, organoids cannot simulate the exchange of gases and nutrients, or the dynamic conditions experienced during vascular or air flow, all characteristics that are essential to the lung. Therefore, organoids may allow studying the cellular response to drugs (*e.g.,* proliferation), but they present limited usefulness for more complex processes including modelling of multi-cellular disease processes and drug metabolism.

Organ-on-a-chip models are another type of tissue engineering system that has been used to recreate, within a microfluidic device, the structure and dynamic microenvironment of lung tissue structures including airways and alveoli. They are attractive because they permit exerting continuous mechanical stimulation and simulating fluid flow and gaseous exchange (Konar et al., 2016; Stucki, 2018). Organ-on-a-chip models allow the study of pharmacokinetics and pharmacodynamics of drugs, both approved and in development, as part of preclinical tests. In addition, organ-on-a-chip models have proven useful in studying the respiration process, but some limitations remain (Konar et al., 2016). For example, it is difficult to maintain long term functionality of cells within microfluidic systems, and complex equipment is needed to monitor the cells (Galimov et al., 2016; Konar et al., 2016; Sundarakrishnan et al., 2018). Although organ-on-a-chip models are elegant systems to study lung tissue interfaces and have proven useful in analyzing how cells behave in the airway and alveoli, the design does not lend itself to the complete reproduction of the 3D nature of tissue, let alone produce a whole organ.

Organ decellularization is another technology with the potential to engineer whole lungs that could be used for modeling lung diseases or for transplantation (Ott et al., 2010; Petersen et al., 2010). Lung decellularization retains most of the lung ECM while removing cells, leaving behind a framework of airway and alveolar structures (Price et al., 2010; Girard et al., 2013). Significant advances have been made in the optimization of decellularization process to maintain the structure and composition of the ECM extracted from animals and humans (Price et al., 2010; Balestrini et al., 2015; Nayakawde 2020; Dabaghi et al., 2021a). However, it is difficult to control cellular distribution during the seeding process and large amounts of cells are required to recolonize the shell of an organ. In addition, effective reendothelization of the pulmonary vasculature remains a challenge and is one of the main causes of failure when trying to recolonize lung ECM scaffolds (Ott et al., 2010; Petersen et al., 2010; Stevens 2011).

The lung models developed have proven useful in studying cell dynamics, disease development and progression, and drug pharmacodynamics and pharmacokinetics. 3D cell culture has shown that introducing a third dimension is key to develop *in vitro* systems that successfully mimic *in vivo* cell microenvironments (Nichols et al., 2014; Bajaj et al., 2016; Galliger, Vogt, and Panoskaltsis-Mortari 2019). However, the main limitation of current models is the strong correlation between structure and function that is characteristic of intact *in situ* systems. The respiratory process subjects cells in the lung to continuous mechanical stress that modulates cell behavior, the expression of ECM components, and impacts healing and disease mechanisms (Doryab et al., 2019). Therefore, to reach the next level in modeling of the lung, it is necessary to develop systems that allow studying the cells in complex 3D designs that resemble the intricate structure and composition of the lung and allow to mimic the continuous mechanical stress that cells are subjected to during the respiratory process. For that, a controlled deposition of different types of cells in the structure is an important aspect. In addition, the materials used to create the scaffolds and cell growth media should present biochemical and mechanical properties that resemble the natural microenvironment that cells encounter in healthy or diseased lung tissue, depending on the desired application. 3D bioprinting is a technology that could address these issues and is therefore a promising tool in the development of truly biomimetic lung models.

## 3D Bioprinting for Tissue Models

Tissue engineering has historically focused efforts on recreating human tissue with two main applications in mind: the design of advanced *in vitro* models for secondary drug screening or toxicology campaigns and the production of functional live tissue for transplantation (Zhu et al., 2016; Derakhshanfar et al., 2018). 3D bioprinting is a technology that could facilitate the construction of *in vitro* tissue to meet both goals. The introduction of the third dimension and the possibility of depositing cells in a controlled manner on engineered supports are advantages of 3D bioprinting technology that are likely to help close the gap between cell culture methods and *in situ* lung tissue. However, printing functional tissues that recreate lung structure and function is still beyond the capabilities of 3D bioprinting technology in 2021.

**FIGURE 2:**
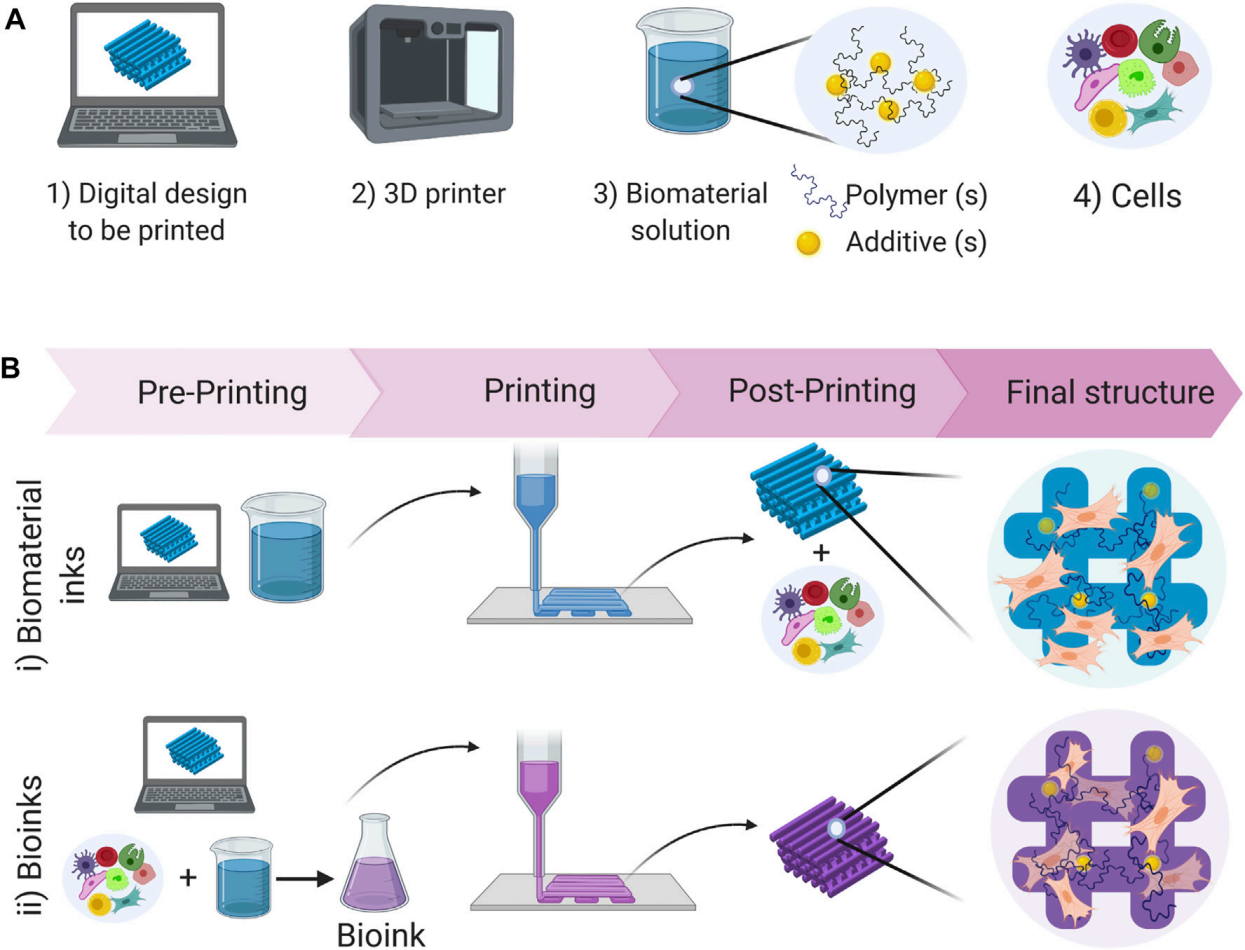
Schematic representation of the bioprinting process: **(A)** Resources required to bioprint structures. **(B)** Comparison of direct bioprinting processes when using biomaterial inks vs bioinks. When using biomaterial inks a post-printing process is required to seed the cells on the structure, which is not required when using bioinks, where the cells are already within the material from the beginning of the process.

The concept of 3D printing was first introduced by Charles W. Hull in 1984 (Hull, 1984), and while it has evolved from that original concept, it remains a bottom-up automated manufacturing process that builds objects from 3D digital designs, often called CAD (computer-aided design) files. 3D bioprinting is the application of this technology to build structures that allow the controlled deposition of biological components in a predetermined design (Figure 2). Depending on the application being developed, the structures can be as simple as a symmetrical scaffold that can support the cells (Berg et al., 2018; Choi et al., 2018). However, there are other applications where the aim is to print more complex constructs and mimic anatomical structures. In such cases, the CAD file can be created based on designed specifications (Ding and Chang 2018; Grigoryan et al., 2019) or can be constructed based on computerized tomography, magnetic resonance imaging or ultrasound imaging (Cheng, 2016; Noor et al., 2019) to reproduce specific features of the organs or tissues for personalized applications.

3D bioprinted structures can be used to mimic the physiological conditions encountered in tissue to study cell behavior under the influence of cell-cell and cell-matrix interactions, cell migration, traction and mechanical stimuli that can be reproduced in the three dimensions. In addition, stiffness or growth factor gradients can be replicated and used to induce adhesion, differentiation, and maturation processes. The crucial factors to be optimized in the application of 3D bioprinting technology are the printing material (including cells and biomaterials used) and the printing protocol (technologies and strategies used during the printing process) (Arslan-Yildiz et al., 2016; Sánchez et al., 2020) (Figure 2). Both aspects are discussed in the following sections.

### Biomaterials and Bioinks for 3D Printing

Cells and biomaterials are the key elements contained in 3D printing inks, but other components (e.g., biomolecules, nanoparticles) (Nguyen and Alsberg 2014; Zhang, Desai, and Ferrari 1998) can be included to improve the biochemical and rheological/mechanical properties of the ink and the printed constructs (Li et al., 2017; Guvendiren and Burdick 2013; Ural et al., 2000; Habib et al., 2018). The printing material can be a biomaterial ink (which does not include cells), or a bioink, which is defined as “a formulation of cells suitable for processing by an automated biofabrication technology that may also contain biologically active components and biomaterials” (Figure 2) (Groll et al., 2018). When using bioinks, the printing process can be done in one step. In contrast, using biomaterial inks requires at least two steps since cells must be seeded after forming the structure. When applying 3D bioprinting to generate structures that can model the lung, deciding whether to include cells in the printable material or not is an important aspect, since it will determine the range of materials, printing and processing conditions that can be used.

The inks used in bioprinting must be non-cytotoxic, present adequate rheological properties so they can be printed, and present structural stability to avoid the printed structure from collapsing under its own weight (Mobaraki et al., 2020). In the case of bioinks, an additional complexity is that the embedded cells must remain viable during and after the printing process. This additional constraint considerably limits the options of materials and reactive chemistries that can be included in the bioink formulation as well as the processing parameters, since high temperatures and pressures will lead to decreased cell viability. Simultaneously fulfilling all these requirements is challenging and the available materials that meet these constraints are presently limited.

**FIGURE 3:**
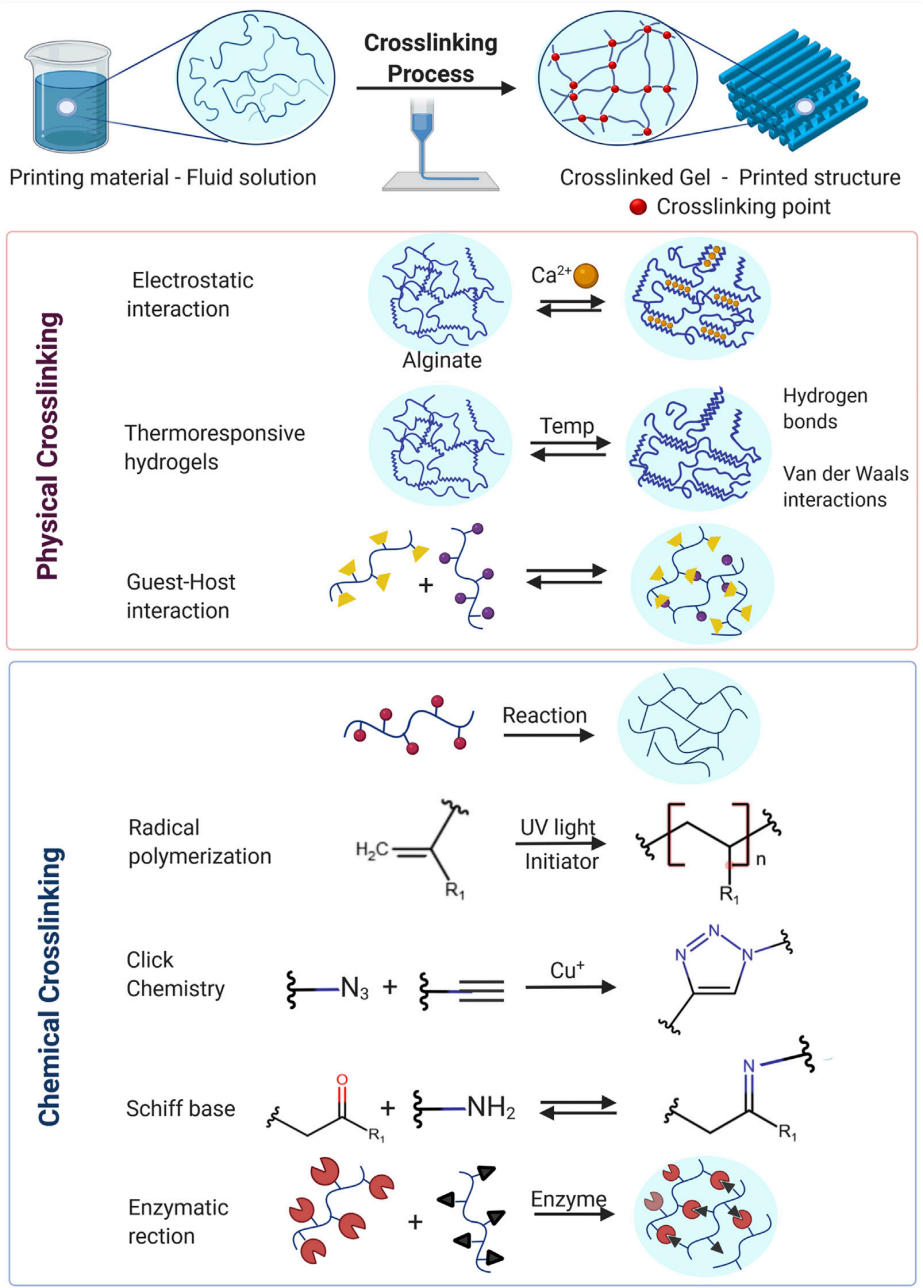
Examples of mechanisms of physical and chemical crosslinking strategies commonly used in 3D Bioprinting.

The materials selected to create 3D lung models are the most important factor in a 3D printing ink because they will determine the parameters during the printing process, the properties of the printed constructs and therefore cell behavior. Crosslinking of the polymeric component in a bioink formulation is the key process that enables the transformation of the viscous liquid into a solid hydrogel capable of retaining the desired shape. The crosslinking process and crosslink density thus determines the mechanical and physicochemical characteristics of the printed structure, which will influence the behavior of the printed cells. Crosslinking mechanisms between polymeric chains can rely on the formation of chemical bonds (covalent crosslinking) (Choi and Cowie, 2019; Wang et al., 2017) or physical interactions like hydrogen bonds, ionic interactions and van der Waals forces (physical crosslinking). Physically or chemically crosslinked hydrogels are the most widely used materials in 3D bioprinting because they swell in aqueous media without dissolving and can therefore offer a fully hydrated environment to the cells (akin to the ECM) that can favor cell survival and proliferation (Sánchez et al., 2020; Zhang and Ali, 2017; Brown and Anseth, 2017). Crosslinking mechanisms commonly used in bioprinting are shown in Figure 3, and more detailed descriptions can be found in comprehensive bioprinting reviews (Hribar et al., 2014; Chia and Wu 2015; Arslan-Yildiz et al., 2016; Yan et al., 2018; Li et al., 2020; Cui et al., 2020).

In the case of using 3D bioprinting for the development of lung models, important aspects frequently overlooked are the mechanical properties of the final constructs. In this regard, photocrosslinkable polymers offer an additional advantage: the crosslink density can be modulated by varying the number of photocrosslinkable moieties in the ink, and by carefully controlling the intensity and time of exposure to UV-light. Higher crosslink density will lead to more robust and stiff constructs (Wang et al., 2017; Choi and Cowie, 2019). Crosslinking of specific components within the bioink is usually achieved during printing, although this process can be started before printing to improve the rheological properties of the bioink or completed after printing to enhance the mechanical properties of the printed structure.

Hydrogels used in bioprinting can be classified as natural biopolymers, like alginate (Park et al., 2017; Aljohani et al., 2018; Habib et al., 2018; Santis et al., 2018; Jeon et al., 2019c), collagen (Kim, Lee, and Kim 2016; Lee et al., 2019; Guo et al., 2021), gelatin (Aljohani et al., 2018; Choi et al., 2018; Tijore et al., 2018), agarose (Daly et al., 2016; López-Marcial et al., 2018), cellulose and cellulose derivatives (Wang et al., 2016; Habib et al., 2018; Li et al., 2020), fibrin (Zhang et al., 2017), hyaluronic acid (Zhang et al., 2017; Kiyotake et al., 2019), and ECM-derived proteins (Pati et al., 2014; Costantini et al., 2016; Santis et al., 2018; Kabirian and Mozafari 2020; Kim et al., 2020; Petrou et al., 2020), or synthetic polymers, like polyethylene glycol (Zhang, Desai, and Ferrari 1998; Alexander et al., 2013; Lee et al., 2014), polyurethane (Lin et al., 2016), polycaprolactone (Shor et al., 2009; Visser et al., 2013; Lee et al., 2014; Jing et al., 2018), and Pluronic F-127 (Noor et al., 2019; Sun and Raghavan 2010). A widely used strategy to produce printable inks is the derivatization of natural biopolymers to introduce reactive functional groups that allow crosslinking mechanisms beyond the naturally occurring ones (García-Lizarribar et al., 2018; Jeon et al., 2019a). For example, GELMA is a derivative of gelatin that has added methacrylate groups that allow thermal or photoinitiated crosslinking of the biopolymer in the presence of a photoinitiator, either during or after the printing process (McBeth et al., 2017; Pepelanova et al., 2018). This additional crosslinking prevents structures printed with GELMA from losing their shape when the temperature is increased to above the gelation temperature of gelatin of 37°C (McBeth et al., 2017; Sun et al., 2018).

The main advantage of using hydrogels made from natural polymers is their high biocompatibility, biodegradability, and the presence of biochemical cues that favor cell adhesion (Wang et al., 2016; Davidenko et al., 2016; Boraschi-Diaz et al., 2017). However, they tend to present poor mechanical properties and high variability between batches, which leads to low experimental reproducibility (Báez, Olsen, and Polarek 2005; Nivison-Smith, Rnjak, and Weiss 2010; Schmidt et al., 2016). In contrast, synthetic polymers present tunable and highly reproducible mechanical properties (Skardal et al., 2015; Hospodiuk et al., 2017; Xin et al., 2019), but lack the biological motifs to promote cell adhesion and pose challenges for enzymatic modification and remodeling. Some research has overcome these limitations through chemical modification or the incorporation of biomolecules (Patterson and Hubbell 2010; Wang et al., 2017; Hosoyama et al., 2019). When trying to create a lung model through 3D bioprinting, experimental reproducibility and cell adhesion to the construct are both important factors that must be taken into account. Given the difficulty to satisfying all the required properties with a single material, mixing different hydrogels in one bioink formulation is a plausible strategy to overcome the intrinsic limitations of each biomaterial while taking advantage of the beneficial characteristics of the mixture (Foss et al., 2013; Aljohani et al., 2018; Cui et al., 2020; Sánchez et al., 2020). In addition, this strategy allows more freedom when trying to modulate the final mechanical properties of the printed construct. For example, alginate/gelatin is a widely studied system that exhibits desired rheological properties, due to the presence of alginate, and an advantageous thermo-responsive gelation mechanism, characteristic of gelatin (Wang et al., 2016; Aljohani et al., 2018; Giuseppe et al., 2018).

Bioinks based on ECM components hold potential for tissue engineering applications, but at the same time they present some of the biggest challenges for 3D bioprinting (Nam and Park 2018; Abaci and Guvendiren 2020; Kabirian and Mozafari 2020). Proteins derived from decellularized tissues show excellent biocompatibility and are ideal materials to fabricate *in vitro* models that mimic natural cell microenvironments because they are naturally derived, can be remodeled by mammalian cells, and present the appropriate biochemical cues and physical characteristics (Frantz, Stewart, and Weaver 2010; Pati and Dong, 2017; Kabirian and Mozafari 2020; Petrou et al., 2020). However, 3D bioprinting the ECM directly is challenging because the decellularization and the enzymatic processes required for gelation result in hydrogels with rheological properties that are not ideal for the extrusion printing process (Pati et al., 2014; Włodarczyk-et al., 2017). This could be one of the reasons why, among the growing number of studies in the field of 3D bioprinting, ECM-derived proteins are still limited (Sayin et al., 2014; Kabirian and Mozafari 2020). Nevertheless, the inherent advantages of using ECM components continue to be strong motivators for the development of new strategies to use them (Pati et al., 2014; Włodarczyk-et al., 2017; Won et al., 2019). To date, extrusion-based printing methods are the most widely used technologies to print ECM (Włodarczyk-et al., 2017; Kabirian and Mozafari 2020). These methods usually take advantage of natural gelation mechanisms or mix the ECM with other polymers to achieve suitable rheological and mechanical properties (Nam and Park 2018; Santis et al., 2018).

Several reports of lung tissue decellularization to obtain ECM derived hydrogels can be found in the literature (Pati et al., 2014; Pati and Dong, 2017; Kabirian and Mozafari 2020; Dabaghi et al., 2021b). There are clear advantages of using lung derived ECM to develop lung models: the composition and the biochemical cues present in the ECM can be easily recognized by the lung cells to be cultured in the development of the model. It has been reported that the mechanical properties of ECM derived hydrogels can resemble the stiffness and viscoelasticity of native lung tissue (Hilster et al., 2020). However, it is difficult to modulate the stiffness of the material without further modification which is the main motivation behind the development of strategies to derivatize the ECM (Petrou et al., 2020). Despite advances in the use of ECM in the formulation of bioinks, there is still little progress in the development of bioprinted lung models using human pulmonary ECM, with the reports available focusing on the use of animal lung-derived ECM to develop bioinks (Santis et al., 2018; Almendros et al., 2019; Tas et al., 2019).

When designing a bioink to implement a lung model, selecting the appropriate cells is another critical aspect given the many options available. The determining factors when selecting cell lines to develop lung models are their characteristics (specific tissue, structure, or disease being modeled) and the objectives of the study (physiological, pathological, toxicological). The primary human cells used as reference are tracheal–bronchial cells and lung alveolar epithelial cells (Ramirez et al., 2004; Fulcher et al., 2005; Hackett et al., 2009; Hoang et al., 2012). Primary cells can be directly isolated from disease-specific lung tissue or healthy donors and can be expanded using standard cell culture methods. For example, our group isolates primary human bronchial epithelial cells from consented subjects undergoing a routine clinical procedure using a bronchial brushing (Dabaghi et al., 2021a; Chandiramohan et al., 2021) as well as primary human lung fibroblasts from lung tissues taken during resection (Dabaghi et al., 2021b). We have demonstrated that these primary cells can be expanded in regular tissue culture plates without losing functionality. It should be noted that primary cells can be difficult to obtain and that complications during the harvesting process can impair their viability. Among the different cell lines available, the A549 human lung carcinoma epithelial cells and the NCI-H441 human papillary adenocarcinoma lung cells are popular cells in modeling pulmonary systems. However, when using transformed cell lines, differences with respect to primary cell behavior are expected because transformed cells derived from human tumors are immortalized, can divide indefinitely, and present a higher growth rate. Cell lines are therefore useful as the first source to study and validate a new bioprinting method that lays the foundation for secondary applications with primary human lung samples from healthy subjects and those with well-phenotyped diseases.

As the lung is a dynamic organ, any bio-printed construct for modeling the lung should be able to undergo constant dynamic forces. These dynamic forces can be in the form of fluid flow or matrix stretching. However, applying dynamic forces to hydrogel-based tissue constructs can be challenging. The integration of microfluidics with hydrogel-based cell culture systems has been proposed as a solution to realize dynamic forces in these systems (Nie, Fu, and He 2020). For example, Abbasi et al. introduced a “pop-it” mechanism to connect hydrogel-based systems with fluidic channels in a reversible fashion (Abbasi et al., 2021). To incorporate mechanical stretching in the system, some researchers used magnetic actuators to oscillate cell-laden hydrogels (Li et al., 2016). To date, specific tissue stretchers have also been designed to integrate oscillation (Shiwarski et al., 2020).

### 3D Bioprinting Technologies

3D bioprinting techniques transform solutions containing polymers, biomolecules, nanoparticles and live cells – in the case of a bioink – into complex 3D structures. This process relies on the crosslinking of the polymeric component which enables the transformation of the liquid, viscous, bioink into a solid hydrogel capable of retaining the desired shape. Each biomaterial presents unique characteristics and specific crosslinking methods are characteristic of certain types of hydrogel, which makes the selection of the printing material a key aspect in the design of the printing process. In addition, not all crosslinking methods can be used with each technology available for bioprinting, which makes matching the crosslinking mechanism with the most appropriate printing technology a necessity. Among the different bioprinting technologies developed, the most promising are inkjet printing, lithography-based printing technologies (e.g., stereolithography and direct laser writing), and extrusion-based printing (Boland et al., 2006; Arslan-Yildiz et al., 2016). A key attribute that sets the different printing techniques apart is the presence or absence of a nozzle, because it determines the workflow of the printing process and the rheological requirements for the printing materials. In the following paragraphs, we present a brief description of each of these 3D bioprinting techniques. The interested reader is referred to existing reviews that have described the different 3D printing techniques in extensive detail (Hribar et al., 2014; Chia and Wu 2015; Arslan-Yildiz et al., 2016; Cui et al., 2020).

Inkjet printing deposits bioink droplets on a substrate through thermal or piezoelectric drop-on-demand delivery methods. This means that the bioinks can be printed without direct contact between the delivering nozzle and the receiving surface, which decreases the risk of contamination. Multiple print heads can be integrated into this type of equipment to facilitate the simultaneous deposition of different bioinks or cell types (Weiss et al., 2005). Inkjet printing enables precise control over droplet deposition and therefore the final location of cells, and it is characterized by high viability (Xu et al., 2005; Xu et al., 2006; Boland et al., 2006; Phillippi et al., 2008; Foresti et al., 2018). Among the limitations of this technology it is important to mention that cells are subjected to thermal and mechanical stress during deposition, which can negatively impact their viability (Gudapati et al., 2016). Additionally, due to the small size of the nozzle the cell types that can be printed are limited and clogging issues are frequent (Gudapati et al., 2016). Another important constraint of inkjet printing technology is that the bioinks must present a relatively low viscosity (*ca.* 100 cP) (Calvert, 2001) which makes the deposition of highly viscous hydrogels and ECM components challenging (Moon et al., 2010). These are important limitations when trying to print constructs for lung models, since some cells might be susceptible to the printing process. Thus, optimizing the rheological properties of the bioinks to satisfy the requirements of the inkjet printing process could be counterproductive when trying to incorporate the cells into the system.

Light-based bioprinting includes a group of technologies that take advantage of photon energy to build scaffold materials and deposit cells in a controlled manner. Different technologies use this approach, like stereolithography (SLA), digital light processing (DLP), continuous digital light processing (CDLP), direct laser writing (DLW), laser-induced forward transfer (LIFT) and volumetric bioprinting. Detailed descriptions about these methods can be found in other reviews (Assenbergh et al., 2018; Pagac et al., 2021; Hon et al., 2008; Hribar et al., 2014; Engelhardt 2013; Zheng et al., 2020; Nguyen and Narayan, 2017; Serra and Piqué, 2019). These technologies use the energy from the laser to initiate polymerization of photo-crosslinkable materials during the printing without the need of a nozzle, which conveys the advantage that high-viscosity bioinks can be used, unlike in inkjet and extrusion-based bioprinting techniques. Specifically, DLW is one of the most popular methods because it provides high resolution and precision, which allows reconstructing tissue details from the millimeter to the sub-micron scale (Barron et al., 2004; Guillotin et al., 2010) but usually requires long printing times. Volumetric bioprinting is a relatively recent and promising technology that overcomes speed limitations of the layer-by-layer printing approach, opening the possibility of printing complex centimeter-scale structures in seconds (Bernal et al., 2019). One of the main drawbacks of this set of technologies when using bioinks is that the laser light and the heat generated can damage cells, affecting viability (Wang et al., 2009; Gudapati et al., 2014; Zheng et al., 2020). A strategy currently being explored to improve cell viability is the development of bioinks that can use lower cytotoxicity visible light during the printing process (Zheng et al., 2020). Other limitations of this approach are the rearrangement of the cells in the precursor solution, specially for long printing times, and the need for photosensitizers or small molecule crosslinking materials that may be cytotoxic (Gudapati et al., 2014). Additionally, since the printing process occurs in a bath of one bioink, controlled deposition of more than one cell type is challenging. These limitations of light-assisted technologies can become major drawbacks when trying to apply this technology to printing constructs to develop lung tissue models that are usually focused on cell behavior and therefore require high cell viability and a strong control over spatial deposition of cells.

Extrusion bioprinting uses a continuous stream of a highly viscous material that is controllably pushed by mechanical force through a nozzle and subsequently gelled or hardened to build a 3D structure. Extrusion printing is one of the most popular methods in biofabrication due to its wide applicability and simplicity (Ji and Guvendiren, 2017). This technology is a promising tool for the development of lung models because it can be used to print materials with a wide range of viscosities with lower risk of clogging (compared to inkjet equipment). Structures with high cell densities and good post-printing viability can be fabricated, but parameters like shear stress during printing must be carefully optimized (Gurkan et al., 2014; Wang et al., 2017b; Yeo and Kim, 2017). The main limitation of this technique is the resolution that can be achieved (∼100 μm) (Zheng et al., 2020), which makes it difficult to recreate detailed tissue features at the micron scale (Arslan-Yildiz et al., 2016; Xiong et al., 2017). Resolution is limited by the nozzle diameter and the rheological properties of the bioink. Optimization of the bioink requires finding the optimum balance between the properties of the bioink to improve shape fidelity (like viscosity, polymer concentration, crosslink density) and maintaining high cell viability. Typically, the conditions that lead to better printing properties lead to worse viability and biological performance of the printed cells, leaving a small window for 3D printing of bioinks containing cells (Schwab et al., 2020).

To deploy the full potential of extrusion bioprinting in creating lung tissue models, it is important to be able to create complex and intricate structures, as well as to print hydrogels that allow cell proliferation, and control over the mechanical properties in the final construct. These are some of the main limitations of extrusion of biocompatible hydrogels, which are usually difficult to print in complex designs due to their soft nature and non-ideal rheological properties. This may also be one the reasons for the slow evolution of the application of extrusion in the development of the lung tissue models. However, to address these limitations new approaches to the extrusion printing process have been explored, and new printing procedures are being developed. These strategies can be useful in the successful application of extrusion for lung model development and are described in the next section.

#### Extrusion Printing Strategies

Extrusion printing is the most widely used technique in bioprinting but is limited by the compromise that must be achieved between the rheological properties of the inks used, the mechanical properties of the printed structure and the parameters selected during the printing process to achieve high cell viability in the final constructs (Schwab et al., 2020). To circumvent the challenges posed by the direct printing of very soft hydrogel materials that afford good cell biocompatibility and viability, researchers have developed assisted bioprinting strategies that can overcome the challenges of direct bioprinting.

**FIGURE 4:**
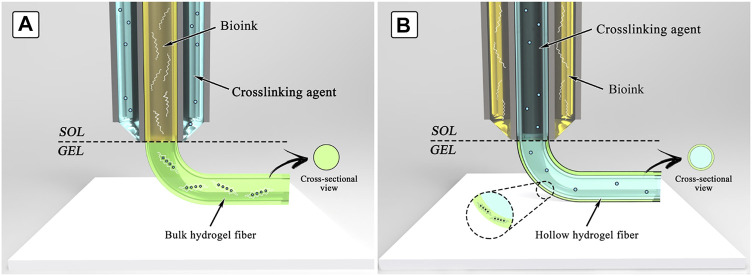
Schematic representation of bioprinting using a co-axial nozzle. Two strategies are possible: **(A)** the bioink is extruded sheathed by a solution of the crosslinking agent, resulting in the formation of a solid fiber; **(B)** the bioink is extruded sheathing a solution of the crosslinker, resulting in the formation of a hollow hydrogel fiber. Figure reproduced from Reference (Costantini et al., 2018).

**FIGURE 5:**
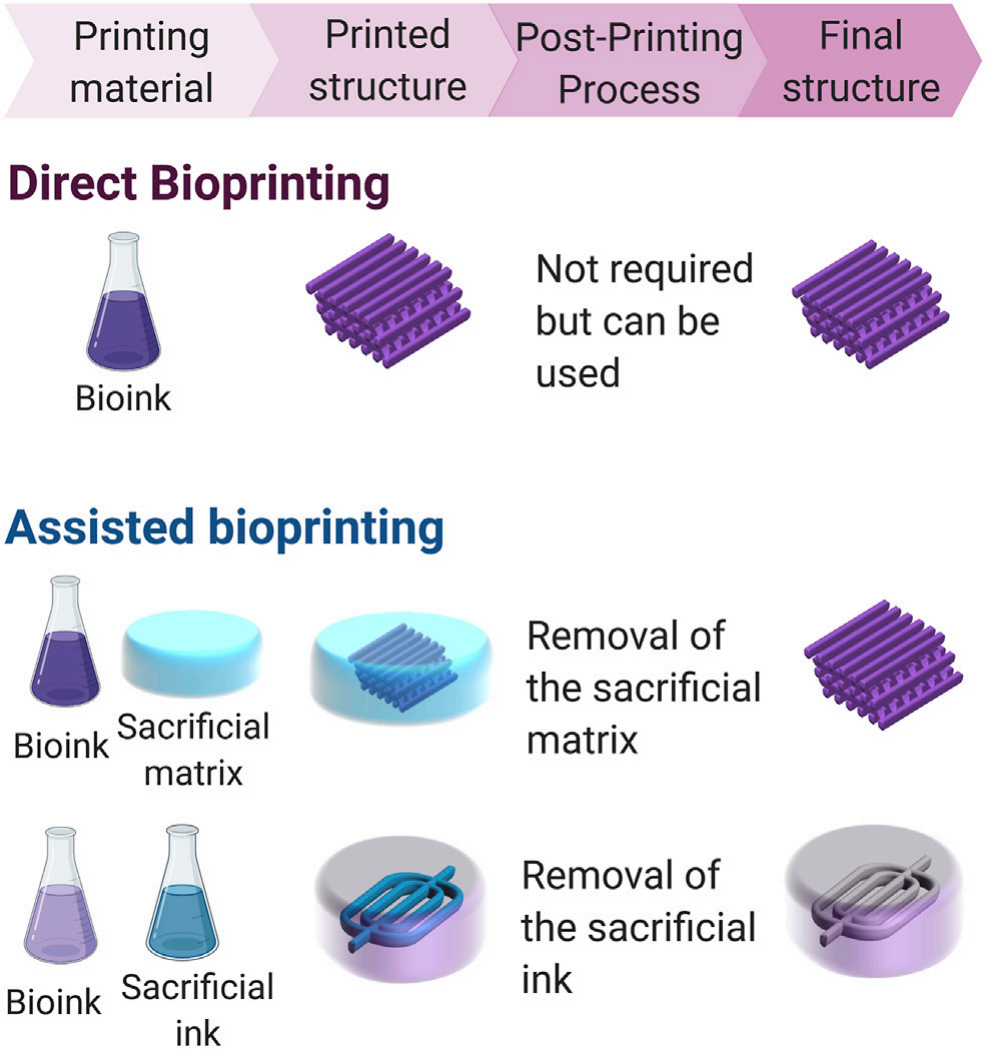
Schematic representation of the strategies applied in bioprinting: direct bioprinting, and assisted bioprinting (using a sacrificial matrix and a sacrificial ink). Direct bioprinting is a straightforward and intuitive approach where the ink is deposited in air to build the desired scaffold. *In situ* crosslinking is required to retain the shape of the construct, and even though the post-printing process is not always needed, it can be used to fine tune the properties of the printed structure. Assisted bioprinting strategies on the other hand take advantage of sacrificial materials to aid during the printing process, allowing for longer crosslinking times. Those sacrificial materials need to be removed after printing to obtain the desired structure.

Direct bioprinting is the intuitive process where materials are deposited on a surface, and the layer-by-layer additive process allows constructing the desired structure (Lee et al., 2020; Zheng et al., 2018; Gu et al., 2020). The crosslinking mechanism determines if additional components need to be added to the ink. For example, if the photocrosslinking mechanism is used, an initiator should be included to allow the crosslinking process to be activated by light. When ionic or small molecule crosslinking agents are needed, the printing process can be done in a bath of a solution containing the crosslinking agent, or the agent can be delivered simultaneously with the bioink by using a coaxial nozzle (Figure 4) (Ozbolat et al., 2014; Zhang et al., 2015; Attalla et al., 2015; Costantini et al., 2018; Colosi et al., 2016; Costantini et al., 2016; Liu et al., 2018). In contrast, assisted bioprinting takes advantage of a support matrix or sacrificial ink to aid the process of printing 3D structures (Figure 5). In both types of assisted bioprinting, the presence of supporting material prevents the collapse of the printed structures and leads to greater structural integrity and fidelity to the original design. Recent reviews have carefully detailed some of the advances made in assisted bioprinting techniques (Chen et al., 2020; Cheng et al., 2020; McCormack et al., 2020).

**FIGURE 6:**
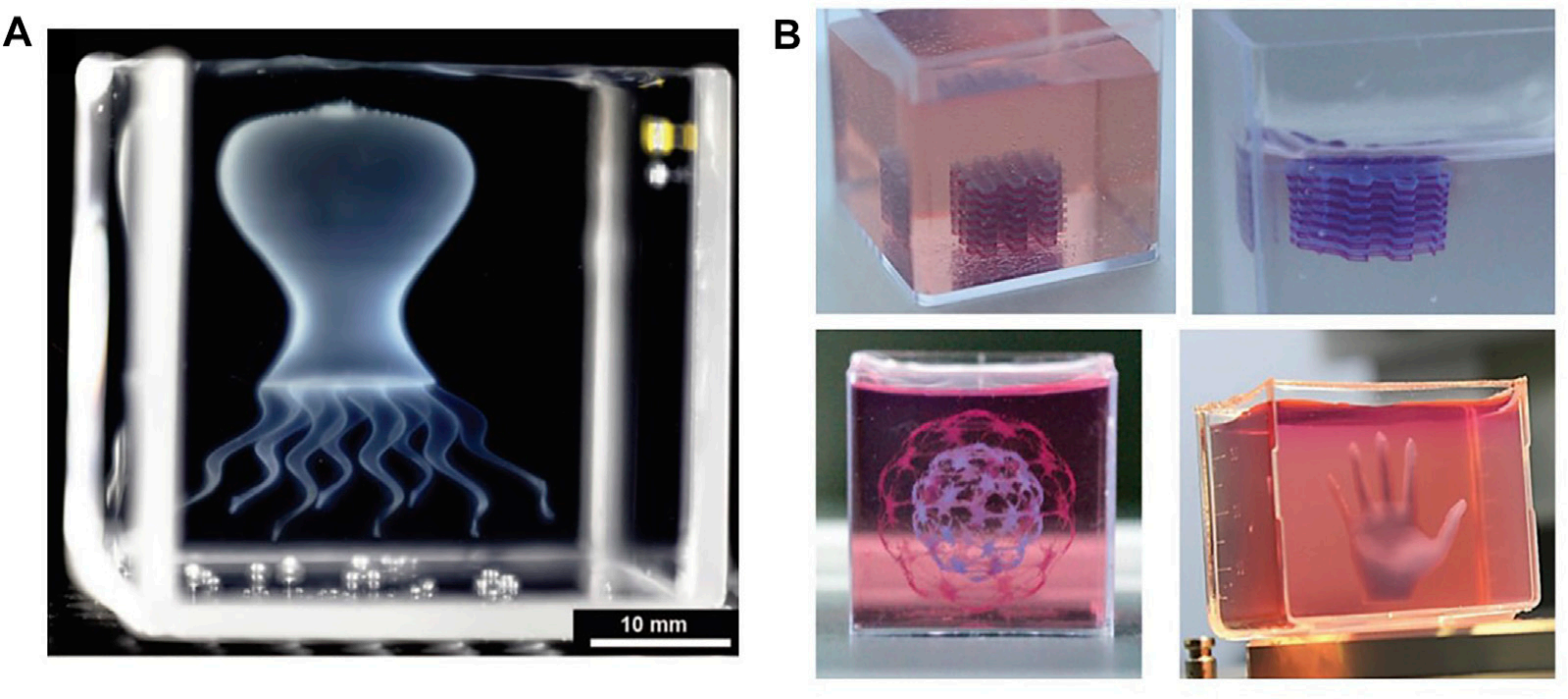
Examples of complex and accurately printed structures using the sacrificial matrix approach. **(A)** Medusa printed as a thin-shell model where multiple hydrogel parts are connected to form the final structure (Reproduced from reference (Bhattacharjee et al., 2015)). **(B)** Complex structures printed inside a support matrix, where the multilayered structure is presented before and after removing the supporting matrix (Adapted from Reference (Noor et al., 2019)).

In assisted bioprinting with a support matrix, the bioink is deposited within the viscous matrix instead of as layers contacting a substrate or surrounded by air (Ning et al., 2020; Li et al., 2020). In addition to giving support to the bioink, the matrix helps retain the printed shape and allows longer crosslinking times (Bhattacharjee et al., 2015; Noor et al., 2019), typically required for thermo-gelling hydrogels (Yao et al., 2017). This strategy also allows true omnidirectional 3D printing, in contrast to layer by layer deposition (or 2.5D printing), which makes it possible to print more complex designs such as those presenting significant overhanging structures. Figure 6 shows examples of complex structures printed using this strategy. This strategy can be very useful when aiming to print complex lung like structures, where a support matrix can aid to recreate the intricate structure of the respiratory tree and help support the structure when the process involves depositing different materials and bioinks with different cells lines. However, for this approach to be successful the matrix must meet specific requirements. First, the matrix needs to give support to the bioink and retain the printed form while allowing the nozzle to move freely to print the structure (Bhattacharjee et al., 2015; Ding and Chang 2018; Noor et al., 2019; Skylar-Scott et al., 2019). Therefore, the support matrix must be made from a material that flows at high shear stress and behaves as a firm solid at low shear stress (Bhattacharjee et al., 2015; Hinton et al., 2016; Ding and Chang 2018; Noor et al., 2019; Li et al., 2020b). Thixotropic polymer solutions (Shi et al., 2017) and granular hydrogels (Cheng et al., 2020; Bhattacharjee et al., 2015) have been extensively used as support matrices because they can be easily optimized to fulfill these requirements (Chen et al., 2020). A second important characteristic is that the material should be easily removed (*i.e.,* sacrificed) without damaging the printed structure in the process (Afghah et al., 2020; Li et al., 2020a). It is also desirable that the material be biocompatible, because any cytotoxic residues retained by the printed structure can damage cells encapsulated within it.

An example of assisted bioprinting using a supporting matrix is the technique called freeform reversible embedding of suspended hydrogels or FRESH (Hinton et al., 2015). This technique allows the use of soft hydrogels that are difficult or impossible to print via direct bioprinting and can be used to produce highly complex structures. The support material used in this process is composed of a slurry of gelatin microparticles that can be easily removed after the printing process by increasing the temperature and washing. Other studies have used similar approaches to print hydrogels lacking ideal rheological properties for printing (Bhattacharjee et al., 2015; Hinton et al., 2016; Noor et al., 2019; Afghah et al., 2020; Li et al., 2020b) and have even successfully printed bioinks composed solely of cells (Jeon et al., 2019b; Jeon et al., 2019c). Additional advantages of this printing approach over direct printing extrusion methods are improved resolution and fidelity when printing hydrogels with poor rheological properties (Bhattacharjee et al., 2015; Tan et al., 2020) and the ability to print hydrogels with slow crosslinking kinetics or poor mechanical properties (Tan et al., 2020). Assisted bioprinting in a support matrix can be an extremely useful strategy to print relevant hydrogels in the creation of human lung models, like collagen and ECM derived hydrogels. This strategy allows more freedom during the printing process and more complex structures can be printed, opening the possibility to print designs that resemble anatomical lung structures. Additionally, crosslinking the structure after completing the printing process creates the possibility of designing more complex printing protocols, making it easier to deposit different cells in a controlled manner inside the structure.

**FIGURE 7:**
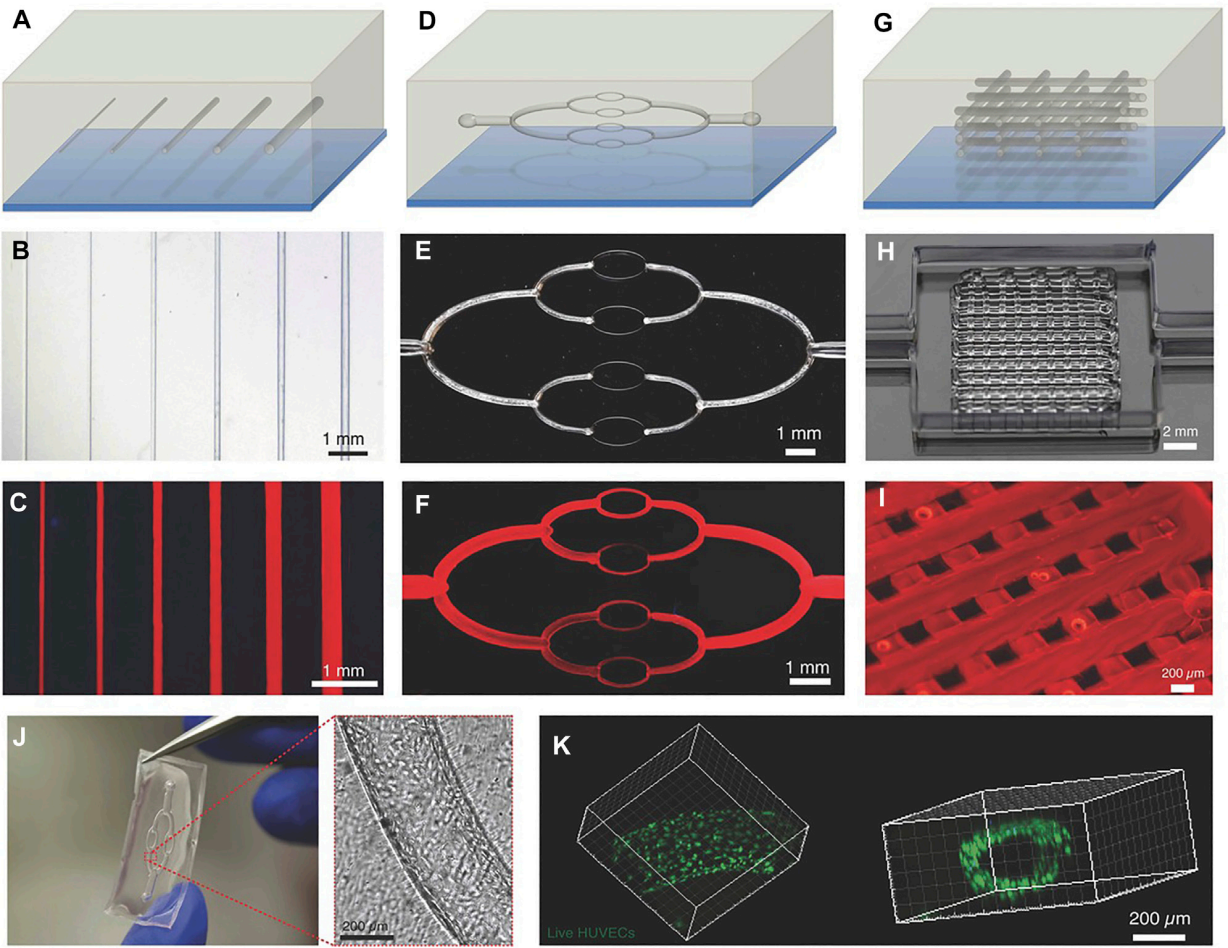
Example of printed structures using sacrificial inks to create channels and vasculature. Schematic illustrations **(A, D, G)**, optical images **(B, E, H)**, and fluorescent images **(C, F, I)** of embedded vascular networks that were printed, evacuated, and perfused with a water-soluble fluorescent dye. Bottom row shows a microchannel that was perfused with HUVEC **(J)** and the corresponding confocal image showing the live cells lining the microcannesl **(K)**. (Reproduced from Reference (Kolesky et al., 2014)).

In assisted bioprinting with sacrificial inks, the bulk matrix is the biomaterial of interest and a structure of the sacrificial ink is printed within it. After the printing process, the sacrificial ink is removed by liquefying and draining it away, while the desired hollow structure remains. Figure 7 shows some examples of 3D printings using sacrificial inks. Sacrificial inks allow to create vasculature in a straightforward way, which is one of the challenges of direct 3D bioprinting (Kolesky et al., 2016; Skylar-Scott et al., 2019; Compaan et al., 2020). Vascularization is a key issue when trying to 3D bioprint lung biomimetic structures because one of the main challenges is to introduce functional vasculature that can support gas exchange. In addition, the concept of removing sacrificial inks post printing could be leveraged to introduce topography beyond the resolution that extrusion technology can offer, which could be helpful when recreating small 3D features like alveoli.

The materials used as sacrificial inks must present good mechanical stability, retain the printed shape and allow efficient printing of complex structures. The presence of channels inside the bulk structure also opens the door to bioprinting larger tissues because the 3D printed channels facilitate the proper exchange of nutrients and waste materials from the cells (Kolesky et al., 2016; Ji, Almeida, and Guvendiren 2019), which requires a maximum distance from the perfusing flow of 100–200 µm (Włodarczyk-et al., 2017) to maintain cell viability. This strategy could also be useful when designing lung tissue models based on extrusion bioprinting because the lung tissue is replete with vasculature. Therefore, a strategy that allows to create intricate structures makes it possible to print features that allow mimicking the interfaces that are present in the lung, which are at the center of the respiratory process.

A variety of polymers can be used as sacrificial materials, with the key requirements being that the material must be easy to extract from the bulk matrix and that the removal procedure must not affect the properties of the printed structure or cell viability. Sacrificial materials can be removed mechanically, a strategy applied for molding structures (Visser et al., 2013), or by using a liquefying or dissolution mechanism (Visser et al., 2013; Ji et al., 2019). Of particular interest are materials with liquefying mechanisms, where disrupting the crosslinked structure of polymers allows them to flow and be removed, a process that can be easily achieved in physically crosslinked polymers. The composition of the sacrificial material determines the liquefying mechanism, with the most common being changing the temperature of thermo-gelling hydrogels and washing the sacrificial material with aqueous solutions (Visser et al., 2013; Bhattacharjee et al., 2015; Kolesky et al., 2016; Ji et al., 2019; Lee et al., 2019). Table 1 shows some examples of materials used in assisted bioprinting and the processes involved in their removal.

**TABLE 1:** Examples of sacrificial materials.

Sacrificial material	Removal procedure	Function	References
Alginate	Wash with citrate solution	Sacrificial ink	(Visser et al., 2013; Jeon et al., 2019a; Noor et al., 2019; Wang et al., 2019)
Polyvinyl alcohol (PVA)	Wash with water	Sacrificial matrix	(Visser et al., 2013; Luo et al., 2019)
Gelatin	Melting gelatin (37°C)	Sacrificial matrix	(Hinton et al., 2015; Lee et al., 2019; Compaan et al., 2020; Heo et al., 2020)
		Sacrificial ink	(Skylar-Scott et al., 2019)
Agarose	Mechanical removal, Wash with water	Sacrificial matrix	(Moxon et al., 2017; Mirdamadi et al., 2019)
Pluronic polymers	Wash with water, Gel to fluid transition (4°C)	Sacrificial ink	(Wu, DeConinck, and Lewis 2011; Kolesky et al., 2014, 2016; Ji, Almeida, and Guvendiren 2019)
		Sacrificial matrix	(Xu et al., 2018; Chiesa et al., 2020)
Carbopol	Wash with saline solution	Sacrificial matrix	(Bhattacharjee et al., 2015; Hinton et al., 2016; Krishnamoorthy, Zhang, and Xu 2019; Ning et al., 2020)

The assisted bioprinting strategies described in this section can be combined in complex printing protocols (Visser et al., 2013; Noor et al., 2019) and can be applied to print different materials sequentially or simultaneously (Colosi et al., 2016; Kaye et al., 2019; Li et al., 2019). Assisted bioprinting techniques allow printing of complex designs that resemble anatomical structures with materials that present poor printability but good cell biocompatibility. These advantages are very useful when trying to create printed constructs to mimic lung structures using materials optimized to create the ideal environment for the cells in the final construct, and therefore are not engineered to satisfy all the rheological requirements for the printing process.

### Modeling the Lung Through 3D Bioprinting

Printing a lung-like structure poses considerable challenges for current 3D bioprinting technology because the lungs are highly complex structures. They are full of vasculature and interfaces that are beyond the resolution or printability that 3D bioprinting technology can offer in 2021. To reproduce the lung microenvironment at the cellular level, both the alveolar epithelium and the vascularization of the lung needs to be reproduced. The native pulmonary blood-air barrier is very thin, on the order of tenths of micrometers, but at the same time robust (Weibel, 2015). To reproduce those characteristics, a minimum spatial resolution of 1–2 microns is required. Although some technologies can achieve such resolution (like multiphoton 3D printing), when using bioinks with non-ideal properties the resolution worsens (Engelhardt, 2013). More importantly, the techniques that reach the required resolution present a limited printing speed (usually below 1 mm/s) making the production of large structures time consuming to a point where it might not be practical. The bronchi, bronchiole, and alveoli not only present complicated geometries to print but also pose the challenge of depositing different cells at specific locations to form functional membranes. The physical properties of lung tissue (*e.g.,* elasticity, gas permeability), which are vital to its function of providing a dynamic cellular environment during the process of breathing, are challenging to mimic with currently available biomaterials and printing processes. An additional complication is that modeling lung diseases requires that the properties of the tissue can be tuned after printing and that cells remain viable until observable changes can be detected and studied.

Over the last few years, significant progress has been made in tuning the mechanical properties of bioinks and printed structures, in improving the control and resolution during and after the printing process, and in developing printing strategies that favor cell viability. Some bioinks using extracellular matrix derived from decellularized lung tissue have been developed, which facilitates mimicking the lung biochemical composition (Santis et al., 2018; Almendros et al., 2019; Tas et al., 2019). However, the advances have not yet had the anticipated impact on the development of 3D printed lung models (Galliger et al., 2019; Skolasinski and Panoskaltsis-Mortari., 2019). To date, applications to lung models have focused on the trachea, showing epithelization and cartilage formation after implantation in animal models (Galliger et al., 2019; Bae et al., 2018; Kaye et al., 2019; Park et al., 2019). Other groups have used 3D bioprinting to model pulmonary diseases and demonstrated advantages over 2D cell cultures. In one instance, the optimization of bioinks to recreate pulmonary infections was reported using combinations of Matrigel^®^, alginate and gelatin (Berg et al., 2018). These systems could reproduce the behavior of viral infections in a more precise way than 2D cells cultures, showing that 3D printing has unexplored potential in the development of pulmonary disease models. In another example, a 3D model for lung cancer was developed to study lung cells for up to 28 days. The model performed better than 2D cultures because the cells exhibited migration and invasion capabilities comparable to those observed in real tissue (Wang et al., 2018).

**FIGURE 8:**
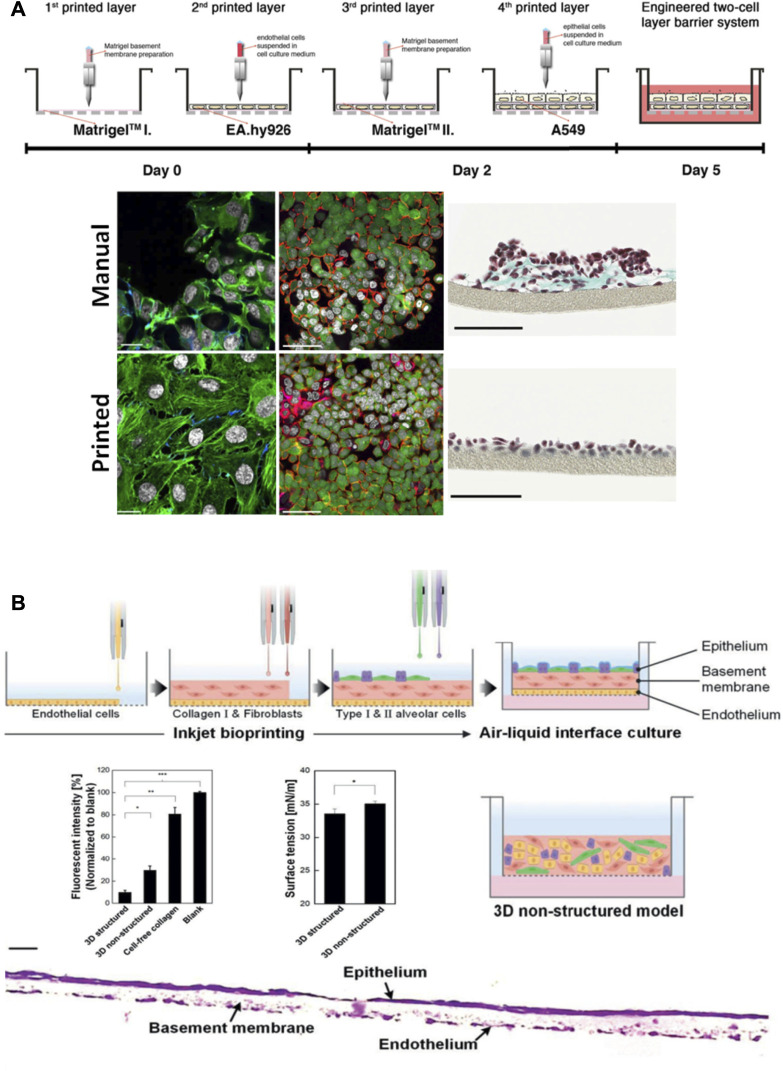
Models that recreate the air-blood barrier by using 3D printing approaches. **(A)** Schematic representation of the process to fabricate an air-blood tissue barrier analog composed of endothelial cell, basement membrane and epithelial cell layers. Comparison of the final cell distribution when seeding cells manually versus bioprinting them: immunofluorescence labelling of F-actin cytoskeleton (green) and nuclei (grey) (scale bar 20 µm), laser scanning micrographs (scale bar 50 µm) and brightfield micrographs (scale bars 100 µm). Reproduced from (Horvath et al., 2015). **(B)** Schematic representation of the fabrication process of an alveolar barrier model composed of alveolar cells, lung fibroblasts, and lung microvascular endothelial cells. Comparison of the 3D model and the 3D unstructured model used as control, and a cross sectional image of the stained barrier (scale bar 20 µm). Reproduced from (Kang et al., 2021).

Another important focus of research has been the development of models to study the air-blood barrier and the alveoli (Horvath et al., 2015; Ng et al., 2021; Kang et al., 2021), which are key to model lung tissue function. Those works have shown how 3D bioprinting is key to control cell deposition to mimic the alveolar barrier (Figure 8). The printing process allowed fabricating functional membranes with improved cell viability and reproducibility, which more accurately mimic the structure and function of the tissue when compared to 2D cell culture models and unstructured 3D systems. Despite the clear advantages of these models to study cell behavior and the resemblance of the printed membranes with the ones present in real tissue, the spatial disposition was planar (Figure 8), and the real 3D globular structure of the alveoli was not fully reproduced. Also, the technologies used to achieve the high resolution required to be able to print such thin features (10–20 µm) (Horvath et al., 2015; Kang et al., 2021; Ng et al., 2021) present low printing speed that limits the practical application of this process to print large volumetric constructs, let alone a full organ.

**FIGURE 9:**
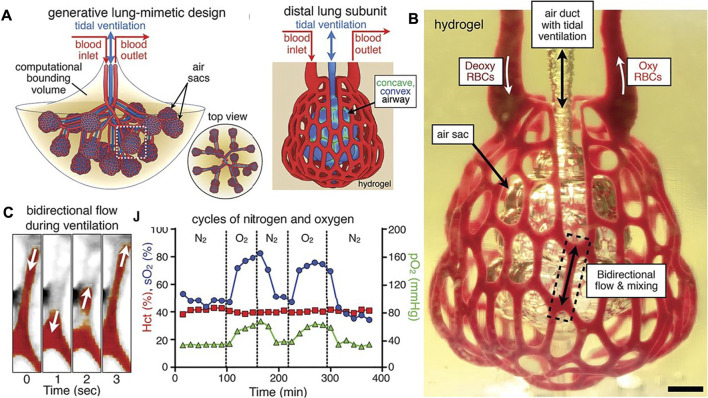
Alveoli-like structures printed using stereolithography (Reproduced from Reference (Grigoryan et al., 2019)). **(A)** Designs that mimic the lung structures where gas exchange occurs as part of the respiration process. **(B)** Printed hydrogel containing the distal lung subunit was perfused with red blood cells in the outside channels while the sac was ventilated with O_2_ (scale bar, 1 mm). **(C)** The printed unit can withstand ventilation cycles, and the model showed sensitivity of the red blood cells to the ventilation gas (N_2_ or O_2_).

Using a completely different approach, a research team successfully printed alveoli-like 3D structures using stereolithography, reducing the depth of irradiation and improving the resolution by adding photoabsorber compounds to the biomaterial ink (Grigoryan et al., 2019). The printed design presented blood vessels that contained oxygenated red blood cells when ventilation was applied and mimicked the rhythmic movement of the pulmonary tissue during the breathing process (Figure 9). This design allowed the researchers to develop a strategy to study the gas permeability of the membrane by perfusing human red blood cells through the printed channels while pumping oxygen and nitrogen through the printed alveoli. Then the perfused blood was collected and analyzed to quantify oxygen and carbon dioxide partial pressure to show successful oxygenation of the fluid. This method sets a precedent to measure air permeability for future works. This research is a significant step forward in the process of printing complex pulmonary structures, but still presented the limitations that the printed design was larger than an actual alveolus and was embedded within a supporting block. Furthermore, the biomaterial ink used was biocompatible but no cells were included, apart from the red blood cells present in the microfluidic blood vessels (Grigoryan et al., 2019).

The works presented so far show that 3D bioprinting can contribute to the process of recreating lung tissue at the cellular and macroscopic level. Tissue constructs present many complex elements and features at microscopic and macroscopic scales. The available 3D printing techniques have different strengths and weaknesses concerning printing speed, resolution, and biocompatibility with cell evolution and maturation. The same applies to other biofabrication techniques, and therefore a recent tendency that has shown promising results is the hybrid approach, where different technologies are applied simultaneously to obtain the desired result (Dalton and Woodfield, 2020). Specifically, 3D printing organoids (Rawal et al., 2021) and 3D printing fabrication of organ-on-a-chip devices (Yu and Choudhury, 2019) show promising results. A bio-printed microfluidic lung-on-a-chip has been reported, made of polycaprolactone with decellularized ECM bioink from tracheal mucosa, that encapsulated endothelial cells and fibroblasts during the printing process (Park et al., 2019). The model recapitulated functional lung tissue structure, like vasculature, where the direct 3D cell printing afforded high reproducibility that could be leveraged during the production of the devices for preclinical trials. Despite the progress in the application of 3D bioprinting in the development of lung models, individually or combined with other biofabrication approaches (Campillo et al., 2021), this area of research is in its infancy and offers a broad range of challenges and opportunities for the development of improved bioinks and 3D printing protocols.

## Challenges and Prospects

Modeling the lung presents unique challenges related to its structure, mechanical properties, dynamic environment, and other important physiological attributes. Many efforts have been dedicated to developing *in vitro* models that allow studying disease evolution and the biodistribution of drugs delivered through the pulmonary route, and significant progress has been made in the development of organ-on-chip models, microtissues, and organoids (Petersen et al., 2010; Nichols et al., 2014; Konar et al., 2016; Gkatzis et al., 2018; Swaminathan et al., 2020). 3D bioprinting offers potential advantages for the development of pulmonary models, but the field is still in the early stages of exploration and optimization to achieve the long-term goal of printing a functional lung. Current efforts in 3D bioprinting are limited because the technology does not yet provide the control or the resolution required to model lung tissue in detail. Nevertheless, the results produced so far show that this technology has an enormous potential that still needs to be unlocked.

Direct bioprinting is a suitable option when the application does not require recreating the cellular microenvironment. In the case of implants, where the most important aspects are biocompatibility, biodegradability and the mechanical properties of the printed structure, along with how it behaves when introduced in the body, optimizing the bioink to meet all these requirements has proven to be a successful strategy (Petersen et al., 2010; Kaye et al., 2019). On the other hand, when trying to create models to study organ function or drug performance, it is ideal that cell behavior can be deliberately modulated to mimic the natural microenvironment by changing the properties of the printed constructs (like the mechanical or biochemical characteristics) (Nichols et al., 2014; Clevers 2016; Fuchs et al., 2002). In these cases, the priority when designing the bioink should be aimed at recreating the natural cell microenvironment while still being able to print the target structure, and the optimization of the bioink to obtain the desired rheological properties should be treated as secondary aspect. To succeed in the printing process while satisfying these opposing requirements, applying more complex protocols than direct bioprinting can be the answer.

So far, the most elegant and advanced 3D bioprinted structures have been obtained by taking advantage of creative strategies while printing, like using sacrificial materials (Visser et al., 2013; Bhattacharjee et al., 2015; Kolesky et al., 2016; Ji, Almeida, and Guvendiren 2019; Lee et al., 2019; McCormack et al., 2020), printing more than one material at a time (coaxial nozzle and multi-bioink printing) (Costantini et al., 2018; Liu et al., 2018; Li et al., 2019), or mixing strategies into one printing protocol (An et al., 2015; Chia and Wu 2015; Zhu et al., 2016; Yan et al., 2018; Li et al., 2019). The last decade has shown us that trying to optimize an ink to create an environment suitable for cells and at the same time obtain appropriate rheological and mechanical properties is challenging and has usually fallen short in one of those aspects (Schwab et al., 2020). The printing strategies described have been successful because they allow to optimize the bioink so the printed structure resembles the natural microenvironment of the cells, and the sacrificial material can be optimized to obtain the required properties that allow a good printing. There will always be room to improve equipment design and bioink properties, but it is important to also pay attention to the printing strategy used.

In this review, several examples are presented where complex structures were successfully printed at high resolution by taking advantage of matrix support and the use of sacrificial inks. Exploring creative ways of combining current developed printing strategies can open a new set of possibilities and allow printing complex constructs that resemble anatomical structures. The use of sacrificial matrices has shown that it is possible to print bioinks with poor printability (Jeon et al., 2019b; Mirdamadi et al., 2019; Chen et al., 2020; Tan et al., 2020), and the use of sacrificial inks has made printing vasculatures feasible (Wu et al., 2011; Kolesky et al., 2014; Ji et al., 2019), one of the biggest challenges of direct bioprinting strategies. These strategies also make it possible to print ECM-based bioinks, which should be explored more in depth for the development of accurate models to study the pulmonary tissue and disease. An additional aspect that needs to be explored in more detail is how cells modify the mechanical properties of the printed structures. The metabolic activity of the cells that populate the construct has the potential to alter the mechanical properties of the material through degradation or the deposition of ECM components. These aspects can be leveraged when designing bioinks for model systems, but further studies are required to understand the interplay between cells and the 3D bioprinted matrices. Another important aspect to consider when designing a model, is the final purpose for which it is being created. Available technologies do not allow yet to print a fully functional lung organ, so deciding which aspects are more relevant to the application that is being developed will help to choose the right technology, printing materials and strategies suitable for the cells to be cultured.

## Conclusion

Advances in tissue engineering are showing that is possible to create reliable *in vitro* lung models. Among the different technologies available, 3D bioprinting shows potential, but much remains to be improved, especially in relation to controlling cell deposition and the resolution of the printed structures. The extensive work and achievements in the field of 3D bioprinting suggest that there is no universal solution to all the challenges bioprinting poses. Each specific application presents requirements that the selected bioink and printing protocol must address. Although the optimization of the bioink is key in the printing process, assisted printing techniques provide additional freedom to the process and therefore, should be considered key players in the design of 3D printing protocols. Lung tissue poses a particular challenge for 3D bioprinting; there is still much to be done to accurately reproduce anatomical shapes, especially for lung tissue that presents such intricate structures. Nevertheless, the results show that it might be possible to achieve this goal by optimizing bioinks and approaching the printing process through a creative lens.
